# Intention to Receive the COVID-19 Vaccine Booster Dose in a University Community in Italy

**DOI:** 10.3390/vaccines10020146

**Published:** 2022-01-19

**Authors:** Lucio Folcarelli, Grazia Miraglia del Giudice, Francesco Corea, Italo F. Angelillo

**Affiliations:** Department of Experimental Medicine, University of Campania “Luigi Vanvitelli”, Via Luciano Armanni 5, 80138 Naples, Italy; lucio.folcarelli@studenti.unicampania.it (L.F.); grazia.miragliadelgiudice@studenti.unicampania.it (G.M.d.G.); francesco.corea@studenti.unicampania.it (F.C.)

**Keywords:** COVID-19, vaccination, hesitancy, willingness, Italy

## Abstract

This cross-sectional study, conducted in Naples (Italy) between 16 November and 6 December 2021, explored the willingness to receive the booster dose of the COVID-19 vaccine among a random sample selected from the list of those who had completed a primary vaccination series at the immunization center of a teaching hospital in Naples and the associated factors. Females had a significantly higher perceived risk of getting the SARS-CoV-2 infection, whereas those not-having a cohabitant were less worried. 85.7% were willing to receive the booster dose. Those older respondents who perceived a better health status after the primary vaccination series, who have friends/family members who were diagnosed with COVID-19, who had received information from official government organizations, and those who did not need information would be willing to get the booster dose. 24.7% was hesitant with a Vaccine Hesitancy Scale (VHS) score ≥ 25. Respondents who self-rated a lower health status after the primary vaccination series, who did not have friends/family members who were diagnosed with COVID-19, who had not received information from official government organizations, and who needed information were hesitant. Information and communication regarding the benefits and efficacy of the booster dose are needed in order to control the pandemic.

## 1. Introduction

The pandemic caused by the new strain of coronavirus (SARS-CoV-2) has affected more than 200 countries and as of 16 January 2022, over 318 million confirmed cases of coronavirus disease 2019 (COVID-19) and 5.5 million deaths had been reported globally and in Italy the total number of cases surpassed 8.2 million while the number of deceased was over 140,000 [1]. It is well-known that universal preventive measures, such as hand washing with soap and water, wearing of face masks, social distancing, covering of the mouth and nose when coughing, and avoiding touching of the face, are the foundation of the pandemic response [2]. Moreover, the availability of vaccines that are effective in preventing symptomatic and asymptomatic SARS-CoV-2 infection has raised hopes of reducing the spread of SARS-CoV-2 infection [3].

In Italy, the COVID-19 vaccination campaign began in December 2020 with prioritization of the most vulnerable populations to the disease, including health care workers (HCWs), residents of nursing homes, the elderly, and essential workers. On 24 March 2021, the vaccination campaign began for extremely vulnerable people, i.e. with severe physical, sensory, intellectual or mental disabilities, and for older people in general [4] and on 4 June 2021, for adolescents aged 12 to 15 years [5]. From 1 December 2021, there was the official recommendation of an additional COVID-19 vaccine booster dose, for people aged 18 years and over at least five months after completing the primary vaccination series [6]. Moreover, from 16 December 2021, it has been recommended an extension of indication for the COVID-19 vaccine Comirnaty (BioNTech/Pfizer) to include use in children aged 5 to 11 [7].

Hesitancy and willingness in relation to the COVID-19 vaccination have been investigated in different groups worldwide with a number of individuals who would either be unwilling to receive it or refuse it altogether despite the severity of the disease [8,9]. However, little is known yet about the intention to receive the booster dose [10,11,12,13,14,15,16]. In this current scenario, this is a challenging issue. Thus, the present exploratory cross-sectional survey was aimed to assess the willingness and the hesitancy to receive the booster dose of the COVID-19 vaccine among a large sample in a university community in Italy. Additionally, the main hypothesis was that respondents were more willing and less hesitant to receive the booster dose if they perceive that they are susceptible to COVID-19, that the disease is severe, and receive information from official government organizations. This article expands on some previous studies, as part of a larger project, of the willingness about this vaccination in a university community [17] and in HCWs [18] and of the impact of the pandemic and vaccination on the behaviors and attitudes of HCWs and students [19].

## 2. Materials and Methods

### 2.1. Setting and Participants

This survey was conducted between 16 November and 6 December 2021, and a total of 1018 potential participants were selected by systematic random sampling from the list of those registered for having received the second dose of the COVID-19 vaccine at the immunization center of a teaching hospital in Naples (Italy) from 26 May to 14 June 2021 and who had not received the booster dose of the COVID-19 vaccine.

A minimum target sample size of 538 was estimated based on the assumption that 70% of the subjects in the population were willing to receive the booster dose of the COVID-19 vaccine, with a margin of error of 5%, a confidence interval of 95%, and considering an expected response rate of 60%.

### 2.2. Procedures

This study was approved by the Ethics Committee of the Teaching Hospital of the University of Campania “Luigi Vanvitelli” (code 1440/2021). The sample received an e-mail invitation with an internet-link leading to a web-based survey platform (Lime Survey). In the invitation letter, participants were informed about the nature and contents of the research, that their participation was on a voluntary basis, that their identity and interviews would remain strictly confidential, that all questions were compulsory, and that they had the right to refuse or withdraw their participation at any time without disclosing a reason. The individual link directed participants to a webpage providing a brief introduction to the objectives of the survey and the access to the online questionnaire. Participants were informed that filling in the questionnaire indicated their agreement to participate in the study. Participants were allowed to respond to the questionnaire only once. Two e-mails were sent and a one-telephone call was made after the initial invitation. No gifts or monetary compensation was provided to participants.

### 2.3. Questionnaire

The questionnaire used in this study was adapted from the contents of instruments that have been used in previously cited surveys [17,18,19]. Piloting of the questionnaire was undertaken among 20 non-selected individuals to evaluate the comprehension of the questions and answers. Those involved in the pre-test were not included in the results.

The questionnaire consisted of 28 questions exploring three domains relating to the respondents: (1) socio-demographic status and general characteristics, including their gender, age, marital status, level of education, number of children in home, having cohabitants, professional role, underlying chronic medical conditions, having been infected with SARS-CoV-2, have had friends or family members who were diagnosed with COVID-19, general self-rated health status and after the first and the second dose of the COVID-19 vaccination; (2) sources of information related to the booster dose of the COVID-19 vaccine they considered reliable and whether they would like to get additional information; and (3) attitudes towards the COVID-19 infection and the booster dose of the COVID-19 vaccine, including the concern that he/she could be infected by the SARS-CoV-2 that was measured on a ten-point Likert scale, where 1 = not at all and 10 = at all, and ten statements towards the COVID-19 vaccination (usefulness, concern about efficacy and safety) on a five-point Likert scale, ranging from 1 = strongly disagree to 5 = strongly agree. Participants were also asked their likelihood to receive or to not receive the booster dose of the COVID-19 vaccine and to select from predefined answers as many reasons as applicable for their decision. The vaccine hesitancy has been evaluated with the 10 items of the Vaccine Hesitancy Scale (VHS) that are measured on a five-point Likert-type scale ranging from ‘strongly disagree’ to ‘strongly agree’ [20].

### 2.4. Statistical Analysis

The data from the questionnaire were analyzed using descriptive statistics, including means and standard deviations for continuous variables and proportions for categorical variables. A series of bivariate analyses were performed to assess the strength of association between each of the independent characteristics and the different outcomes of interest by using the chi-square test and Student’s *t*-test, respectively, for the categorical and for the continuous variables. Those independent characteristics that had a *p*-value less than or equal to 0.25 in the bivariate analyses were further incorporated into the multivariate linear and logistic regression models. Three multivariate models were designed to determine the independent associations between predictors and the following outcome variables: perceived risk of being infected by SARS-CoV-2, which was measured with a value ranging from 1 “low” to 10 “high” (Model 1); willingness to receive the booster dose of the COVID-19 vaccine, which was dichotomized as 1 if the answer was “yes” and 0 if the answer was “no” or “uncertain” (no = 0; yes = 1) (Model 2); and booster dose COVID-19 vaccine hesitancy (VHS score <25 = 0; VHS score ≥25 = 1) (Model 3). The following independent variables have been tested because potentially related to all outcomes gender (male = 0; female = 1), age, in years (continuous), marital status (unmarried/separated/divorced/widowed = 0; married/cohabited with a partner = 1), having cohabitants (no = 0; 1–3 = 1; >3 = 2), baccalaureate/graduate degree (no = 0; yes = 1), role (student = 0; other = 1), having at least a chronic medical condition (no = 0; yes = 1), having been infected with SARS-CoV-2 (no = 0; yes = 1), have had friends or family members who were diagnosed with COVID-19 (no = 0; yes = 1), self-rated global health status (continuous), self-rated health status after the first dose (continuous), self-rated health status after the second dose (continuous), having received information on the booster dose of the COVID-19 vaccine from official government organizations (no = 0; yes = 1), and need of additional information on the booster dose of the COVID-19 vaccine (no = 0; yes = 1). A stepwise method was used to retain or to exclude in the final multivariate models the variables with a threshold of *p* = 0.2 and *p* = 0.4, respectively. Results of the logistic regression models were measured using Odds Ratios (ORs) together with their 95% confidence intervals (CIs), whereas results of the linear regression models using standardized regression coefficients (ß). All analyses were based on two-sided *p*-values, with statistical significance defined as *p* equal to or less than 0.05. The statistical analysis was conducted with the use of STATA, version 15.1 [21].

## 3. Results

### 3.1. Characteristics of the Respondents

Of the total 1018 subjects selected, 761 opened the survey and 146 of them were excluded because they failed to respond to 12 questions, leaving a final sample of 615 valid responses with a response rate of 60.4%. Table 1 provided the socio-demographic and key characteristics of the study population. Participants had a mean age of 32.1 years, more than half were female (57.4%), nearly three-quarters (77.4%) were unmarried/separated/divorced/widowed, more two-thirds were students (71.1%) and had a high school degree or less (69.3%), only 11.9% had at least one chronic medical condition, almost 10% have been infected with SARS-CoV-2, and the vast majority (89.8%) have had friends or family members who were diagnosed with COVID-19.

### 3.2. Attitude towards COVID-19

Regarding the attitudes towards the COVID-19 disease and the booster dose of the COVID-19 vaccine, the self-reported risk perception of getting the infection, measured on a 10-point Likert-type scale, resulted in a mean value of 6.8 ± 2.3, with the 3% and 15.2% of the respondents who believed that the risk 1 and 10, respectively. Multivariate linear and logistic regression analyses were performed to investigate the predictors that were associated with the different outcomes of interest in the bivariate analysis with a *p*-value of smaller than 0.25 and the results are shown in Table 2. Only two variables remained associated with the self-reported risk perception of getting the infection after multivariate linear regression. Female had significantly higher levels of risk perception about COVID-19, whereas those not having cohabitant, compared with those who had no more than three cohabitants, were less worried of the SARS-CoV-2 infection (Model 1).

The majority of the sample (85.7%) reported that they were willing to be vaccinated against COVID-19 with the booster dose, whereas 2.3% and 12%, respectively, were unwilling and uncertain. Model 2 showed the odds ratios for participants willing to receive the booster dose of the COVID-19 vaccine and the results showed that of all baseline characteristics of the participants, only age was associated, with those older that reported that they would be willing to get the booster dose (OR = 1.03; 95% CI = 1.01–1.07). Moreover, the willingness has been observed in respondents who perceived a better health status after the two doses of the vaccine (OR = 1.22; 95% CI = 1.07–1.40), those who have friends or family members who were diagnosed with COVID-19 (OR = 2.00; 95% CI = 1.02–3.92), those who had received information about the booster dose from official government organizations (OR = 1.68; 95% CI = 1.03–2.73), and those who did not need additional information about the booster dose (OR = 0.46; 95% CI = 0.28–0.74) (Model 2 in Table 2). The main reasons why participants were willing to receive the dose were because they would protect themselves (72.6%) and their relatives (65.8%). Among those who were unwilling or uncertain to get the booster dose, the top two common reasons reported were the concern about the safety (43.2%) and belief that the protection against SARS-CoV-2 infection has already been acquired after a two-dose schedule of COVID-19 vaccines (27.3%).

The details for respondents’ answers regarding the hesitancy toward the booster dose of the COVID-19 vaccine measured using the VHS index are shown in Table 3. The overall mean VHS score was 20.4 ± 5.8 and about one-fourth of the sample (24.7%) was considered hesitant with a score ≥ 25. One in four and 16.7% respondents disagreed or were undecided whether the booster dose is effective and useful, respectively. More than half was concerned or uncertain about serious side effects and only 10.9% disagreed or were uncertain that the dose is important for the health of others in the community. Overall, 75.1% of the sample responded in a hesitant way to at least one of the 10 items and 2.4% responded hesitant to all ten items.

Results of the multivariate logistic regression model showed that respondents who self-rated a lower health status after the two doses of the COVID-19 vaccination (OR = 0.82; 95% CI = 0.69–0.98), those who did not have friends or family members who were diagnosed with COVID-19 (OR = 0.35; 95% CI = 0.20–0.63), those who had not received information about the booster dose from official government organizations (OR = 0.49; 95% CI = 0.32–0.73), and those who needed additional information about the booster dose (OR = 2.29; 95% CI = 1.54–3.41) were more likely to be hesitant toward the booster dose of the COVID-19 vaccine with a VHS value > 25 (Model 3 in Table 2).

### 3.3. Sources of COVID-19 Vaccine Booster Dose Information

Almost all respondents reported a variety of sources of information about the booster dose of the COVID-19 vaccine (99.7%). Among sources, multiple answers were possible, used to acquire information, more than half of respondents ranked physicians as highly trustworthy source (57.8%), followed by official government organizations (48.6%), Internet (38.2%), and mass-media (36.4%). Of note, less than half (44.6%) of the participants said that they would like to acquire more information related to the booster dose of the COVID-19 vaccine.

## 4. Discussion

To the best of our knowledge, this survey is among the few that have investigated the willingness to accept the COVID-19 vaccine booster dose as well as the potential facilitators and barriers that may influence their decision among a university community in Italy. Several important findings arose from the current survey.

First, a large proportion of those who took part in the current survey (85.7%) reported that they were willing to receive the COVID-19 vaccine booster dose. A notable finding was that the hesitancy towards the booster dose was low among the responders since 24.7% of the sample were classified as hesitant with a total VHS score ≥25. Other surveys have found varying proportions of participants willing to receive the booster dose with values of 55.3% among HCWs in Saudi Arabia [13], 61.8% in adult Americans [14], 71% among adults in Poland [10], 71.3% among HCWs in Czechia [15], 83.6% a hypothetical yearly booster vaccine among healthcare workers in the United States [12], 84.5% among medical students in Japan [11], and 91.1% in the general population in China [16]. In the present study interviewees highlighted that the most common reasons in vaccine-related decision-making were because they would protect themselves and their relatives. One finding that merits highlighting is that the answers of respondents to the open question behind unwillingness or uncertainties to receive the booster answers revealed a wide range of concerns. Indeed, the respondents’ beliefs that the booster dose was unsafe and that the protection against SARS-CoV-2 infection has already been acquired after a two-dose schedule of COVID-19 are striking. Two recent meta-analyses of studies in the real-world setting have shown that the approved vaccines have reassuring safety and are highly protective against SARS-CoV-2 [22,23]. Moreover, a phase 2 trial of third dose booster vaccines showed acceptable side-effect profiles and the immunogenicity of homologous or heterologous third dose was superior to control regardless of which vaccine had been received in the initial course [24]. Thus, a better-organized public health program and educational policies to disseminate clear and credible information about the booster dose must be implemented and this could have an important impact in addressing the fears about the safety of the vaccine. Widespread sensitization is needed to promote the understanding of the safety of COVID-19 vaccination and this is also supported by the fact that almost half of the respondents pointed out that they would like to get additional information. Furthermore, an interdisciplinary approach with the contribution of experts from various fields is needed to develop multiscale framework to shed light on the spread of pandemics and somehow motivated also by care both for individuals and society as a whole [25].

Second, it is worth noting to mention that the results of this survey provide important insights into the main sources commonly utilized to obtain information related to the booster dose of the COVID-19 vaccination. Participants stated that physicians and official government organizations were the principal sources of information. Official government organizations can be used effectively to provide reliable, credible, updated, and useful information about COVID-19 vaccination since they are highly trusted by the general public. The results of the study showed that being informed by official government organizations was associated with greater odds of willingness to receive the booster dose of the COVID-19 vaccine, whereas those who did not acquire information from this source were more likely to be hesitant. This finding is of particular significance and it is in line with those from previous literature among different groups of individuals illustrating that official government organizations, HCWs, and scientific journals are recognized as key in addressing individuals’ health issues and motivating them to increase preventive activities utilization. Indeed, these sources have been identified as an important influential factor to individuals’ level of adequate knowledge, positive attitudes, and high vaccine uptake [18,26,27,28,29,30]. Therefore, these sources should provide scientific information about the pandemic to generate awareness regarding the importance of preventive measures and in influencing decisions about whether or not to be vaccinated, such as for the booster dose of the COVID-19 vaccine. However, it should be noted that more than one-third of the respondents reported seeking this information from Internet. Receiving health information from Internet may be an easier source, but concerns should be raised since misinformation and lack of accurate medical knowledge about the pandemic could be disseminated rapidly through this source without supervision with a potentially negative influence on knowledge, attitudes, and behavior. Furthermore, less than half of all participants said that they would like to acquire more information related to the booster dose of the COVID-19 vaccine. An interesting observation was that those who did not need additional information about the booster dose of the COVID-19 vaccine were more willing to receive the booster dose and, by contrast, those who needed information were more likely to be hesitant. This latter finding provides insights into the necessity of efforts focused on educational programs by providing adequate information of the booster dose to ensure the adherence of the individuals to the vaccination strategy during the COVID-19 pandemic.

Third, the multivariate linear and logistic regression analysis showed that few socio-demographic and general characteristics significantly explained the different outcomes of interest. Indeed, females perceived a higher risk that they can be infected by the SARS-CoV-2, whereas those respondents with no cohabitants were less worried about the risk of being infected with the virus. The association with the gender is in accordance with previous studies across different countries [10,31,32,33] and may be explained by the fact that the spread of the COVID-19 in Italy has affected females slightly more than males, although the mortality rate was higher for males. Moreover, those older were more likely to accept the booster, which is consistent with prior studies on this vaccination [10,12,34]. This finding is not surprising and it may be because of the fact that older people are at higher risk of severe disease from infection of COVID-19. Finally, respondents with friends or family members who were diagnosed with COVID-19 exhibited more willingness to receive the booster dose, whereas those who did not have such experience were more likely to be hesitant. This association is expected, since the willingness can develop in people after they have experienced or witnessed COVID-19 that threatens their health or, as in the present study, that of others around them. This finding has been reported in previous studies conducted in other parts of the world [35,36].

This survey recognizes few potential methodological limitations that should be borne in mind when interpreting these results. Firstly, caution should be taken when interpreting the findings owing to the cross-sectional design of the survey that did not allow confirming the final temporal and the causal inferences to associations between the independent variables and the outcomes of interest. Secondly, the survey was undertaken in a single center, thus the responders cannot be taken as fully representative of the attitudes of the population in other regions of Italy. Thirdly, it should not neglect that the respondents may have given socially desirable responses. However, the complete anonymous feature of the survey has been emphasized to participants and in this way the influence of desirability bias should be reduced. Despite these limitations, the survey offers useful information for policymakers and HCWs on this important topic.

## 5. Conclusions

In conclusion, the findings arising from this survey provide important information regarding the hesitancy and the willingness to accept the booster dose of the COVID-19 vaccine. It should be emphasized that is crucial to provide information and communication regarding the benefits and the efficacy of the booster dose, especially as the number of COVID-19 cases still exists, in order to control the pandemic.

## Figures and Tables

**Table 1:** Socio-demographic and key characteristics of the study population.

Characteristics			N	%
*Age, Years*			32.1 ± 15.9 (19–76) *	
Gender				
Female			353	57.4
Male			262	42.6
Marital status				
Unmarried/Separated/Divorced/Widowed			476	77.4
Married/Cohabited with a partner			139	22.6
Education level				
High school degree or less			426	69.3
Baccalaureate/Graduate degree			189	30.7
Number of children in home				
0			511	83.1
≥1			104	16.9
Number of cohabitants				
0			32	5.2
1–3			417	67.8
>3			166	27
Role				
Student			437	71.1
Other			178	28.9
Having at least a chronic medical condition				
No			542	88.1
Yes			73	11.9
Having been infected with SARS-CoV-2				
No			556	90.4
Yes			59	9.6
Having friends or family members who were diagnosed with COVID-19				
No			63	10.2
Yes			552	89.8
Self-rated global health status			8.3 ± 1.3 (1–10) *	
Self-rated health status after the first dose of the COVID-19 vaccination			8.1 ± 1.5 (1–10) *	
Self-rated health status after the second dose of the COVID-19 vaccination			8.1 ± 1.5 (1–10) *	

* Mean ± Standard deviation (range).

**Table 2:** Multivariate linear and logistic regression analysis results examining the outcomes of interest according to several explanatory variables.

Variable	Coeff.	SE	*t*	*p*
Model 1. Perceived risk of being infected by SARS-CoV-2 F (4, 610) = 13.93, *p* < 0.0001, R^2^ = 8.37%, adjusted R^2^ = 7.77%				
Female	1.23	0.18	6.62	<0.001
Number of cohabitants				
0	−0.99	0.41	−2.39	0.017
1–3	1.00 *			
>3	−0.27	0.2	−1.33	0.186
No need for additional information regarding the booster dose of the COVID-19 vaccine	0.31	0.18	1.68	0.093
	OR	SE	95%CI	*p*
Model 2. Willingness to receive the booster dose of the COVID-19 vaccine Log likelihood = −233.34, χ^2^ = 38.26 (7 df), *p* < 0.0001				
No need for additional information regarding the booster dose of the COVID-19 vaccine	0.46	0.11	0.28–0.74	0.002
Higher self-rated health status after the second dose of COVID-19 vaccination	1.22	0.08	1.07–1.40	0.003
Having received information regarding the booster dose of the COVID-19 vaccine from official government organization	1.68	0.41	1.03–2.73	0.034
Older	1.03	0.01	1.01–1.07	0.042
Having friends or family members who were diagnosed with COVID-19	2.00	0.68	1.02–3.92	0.043
Having at least a chronic medical condition	1.66	0.75	0.68–4.04	0.258
Student	0.63	0.32	0.23–1.71	0.372
Model 3. Booster dose COVID-19 vaccine hesitancy Log likelihood = −310.68, χ^2^ = 66.43 (8 df), *p* < 0.0001				
Need of additional information regarding the booster dose of the COVID-19 vaccine	2.29	0.46	1.54–3.41	<0.001
Not having friends or family members who were diagnosed with COVID-19	0.35	0.10	0.20–0.63	<0.001
Not having received information regarding the booster dose of the COVID-19 vaccine from official government organization	0.49	0.10	0.32–0.73	0.001
Lower self-rated health status after the second dose of COVID-19 vaccination	0.82	0.07	0.69–0.98	0.032
Married/cohabited with a partner	1.48	0.35	0.92–2.37	0.102
Number of cohabitants				
0	1.62	0.69	0.70–3.77	0.255
1–3	1.00 *			
>3	1.40	0.32	0.89–2.21	0.136
Lower self-rated health status after the first dose of COVID-19 vaccination	0.90	0.08	0.76–1.07	0.265

* Reference category.

**Table 3:** Descriptive characteristics of respondents’ VHS index about the booster dose of the COVID-19 vaccine.

Item	Participants’ Response	N	%
The booster dose of COVID-19 vaccine is important for my health	Disagree	14	2.2
	Not sure	76	12.4
	Agree	525	85.4
The booster dose of COVID-19 vaccine is efficacy	Disagree	10	1.6
	Not sure	151	24.5
	Agree	454	73.9
It’s important getting the booster dose of COVID-19 vaccine to protect you and those around you	Disagree	13	2.1
	Not sure	54	8.8
	Agree	548	89.1
The booster dose of COVID-19 vaccine is useful	Disagree	12	1.9
	Not sure	91	14.8
	Agree	512	83.3
The booster dose of COVID-19 is more dangerous than the first and the second dose	Disagree	327	53.2
	Not sure	242	39.3
	Agree	46	7.5
The information I receive from the Ministry of Health on the booster dose of the COVID-19 vaccine is reliable	Disagree	27	4.4
	Not sure	142	23.1
	Agree	446	72.5
Getting the booster dose of the COVID-19 vaccine is an effective strategy to protect me from the disease	Disagree	14	2.3
	Not sure	92	14.9
	Agree	509	82.8
I follow my doctor’s advice about the booster dose of the COVID-19 vaccine	Disagree	20	3.2
	Not sure	86	14
	Agree	509	82.8
I am worried about a serious side effect after getting the booster dose of the COVID-19 vaccine	Disagree	272	44.2
	Not sure	183	29.7
	Agree	160	26.1
I don’t need the booster dose of the COVID-19 vaccine	Disagree	447	72.7
	Not sure	133	21.6
	Agree	35	5.7

## Data Availability

The data presented in this study are available on request from the corresponding author.
